# Respiratory Syncytial Virus Infection Triggering a Pulmonary Hypertensive Crisis in a Boy With Prader–Willi Syndrome-Associated Sleep-Disordered Breathing

**DOI:** 10.7759/cureus.103890

**Published:** 2026-02-19

**Authors:** Mari Tsukahara, Hideaki Yagasaki, Koichi Makino, Tomoaki Sano, Takeshi Inukai

**Affiliations:** 1 Department of Pediatrics, Faculty of Medicine, University of Yamanashi, Yamanashi, JPN

**Keywords:** growth hormone therapy, pediatric obstructive sleep apnea, prader-willi syndrome, respiratory syncytial virus (rsv), sleep-disordered breathing

## Abstract

Prader-Willi syndrome (PWS) is a multisystem genetic disorder frequently complicated by sleep-disordered breathing, including obstructive sleep apnea. Respiratory infections may precipitate acute decompensation in vulnerable patients with PWS. A four-year-old boy with genetically confirmed PWS receiving growth hormone therapy developed respiratory distress and cyanosis. He was found to be respiratory syncytial virus-positive and presented with acute respiratory acidosis and markedly elevated aminotransferases with coagulopathy. Echocardiography showed right-sided cardiac dilation consistent with pulmonary hypertension. He was managed with noninvasive ventilation, oxygen supplementation, and diuretics, followed by step-down to nasal continuous positive airway pressure. Liver enzymes normalized as his respiratory status improved. Polysomnography after stabilization showed obstructive sleep apnea with considerable nocturnal desaturation, and home continuous positive airway pressure was initiated.

Respiratory syncytial virus (RSV) infection can trigger acute-on-chronic respiratory failure and cardiopulmonary decompensation in children with PWS and unrecognized severe nocturnal hypoxemia. Early screening and ongoing surveillance for sleep-disordered breathing, cardiopulmonary complications, and careful management during intercurrent infections are essential. RSV immunoprophylaxis, such as palivizumab, may warrant individualized consideration in high-risk young children with PWS who have impaired airway clearance or considerable respiratory comorbidity.

## Introduction

Prader-Willi syndrome (PWS) is a complex genetic disorder caused by the loss of expression of paternally inherited genes in the 15q11.2-q13 region. PWS is characterized by hypotonia, developmental delay, endocrine dysfunction, and progressive hyperphagia leading to obesity [1]. Sleep-disordered breathing (SDB) is common and clinically significant in individuals with PWS across the lifespan [2]. The spectrum of SDB in PWS includes central sleep apnea and/or obstructive sleep apnea (OSA) driven by upper airway narrowing, obesity, hypotonia, and abnormal ventilatory control and arousal responses [3]. Pulmonary hypertension and cardiopulmonary complications have been reported in patients with PWS, particularly in association with severe SDB and chronic hypoventilation, and may contribute to significant morbidity and mortality [4]. Growth hormone (GH) therapy improves body composition and linear growth in children with PWS. However, concerns remain regarding the potential for worsening upper airway obstruction in some patients with PWS, indicating the importance of baseline and follow-up polysomnography [5,6].

Respiratory syncytial virus (RSV) is a leading cause of lower respiratory tract infection in infants and young children worldwide and is a major contributor to hospitalization for bronchiolitis and pneumonia [7]. While RSV infection is often self-limited in otherwise healthy children, it may result in severe respiratory compromise in patients with underlying conditions. These conditions include chronic lung disease, neuromuscular disorders, and congenital syndromes associated with impaired airway clearance or ventilatory control [8]. We report a boy with PWS who developed acute respiratory failure accompanied by a pulmonary hypertensive crisis and marked transaminase elevation following RSV infection. A subsequent evaluation showed clinically significant OSA with profound nocturnal desaturation.

## Case presentation

**Figure 1:**
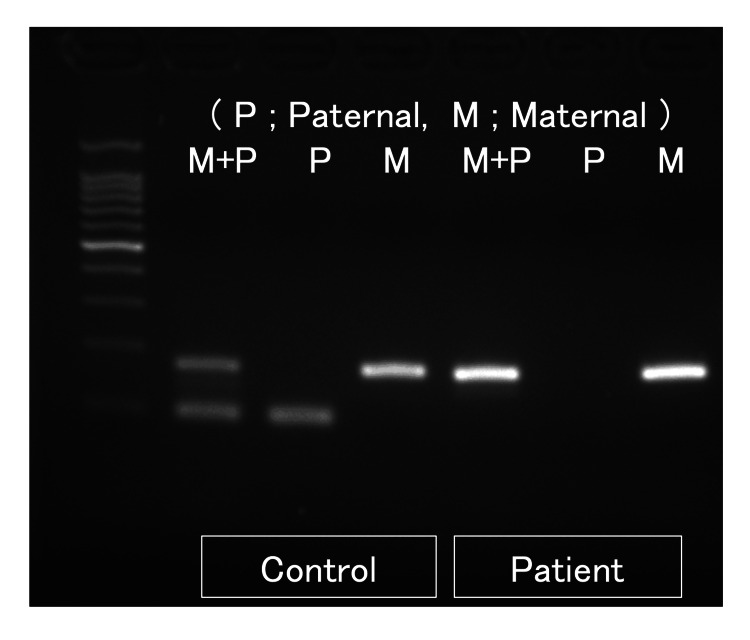
Methylation-specific polymerase chain reaction analysis of the 15q11.2–q13 region. Methylation-specific polymerase chain reaction (MS-PCR) was performed to evaluate the methylation status of the Prader–Willi syndrome critical region. In the control sample, paternal (P) and maternal (M) alleles were detected, as shown by the presence of amplification products in the respective lanes. In contrast, the patient sample showed amplification of the maternally methylated allele only, with absence of the paternally derived allele. These findings are consistent with the loss of expression of paternally inherited genes in the 15q11.2–q13 region, confirming the molecular diagnosis of Prader–Willi syndrome.

A four-year-old boy with PWS was transferred to our tertiary center for acute respiratory failure. He was born at 37 weeks and four days of gestation, weighing 2,042 g. In the neonatal period, he showed hypotonia, poor feeding, failure to thrive, micrognathia, low-set ears, and cryptorchidism. Fluorescence in situ hybridization did not show a deletion in the 15q11.2-q13 region. Methylation-specific polymerase chain reaction demonstrated the absence of the paternally inherited allele, which confirmed the diagnosis of PWS (Figure 1).

**Figure 2:**
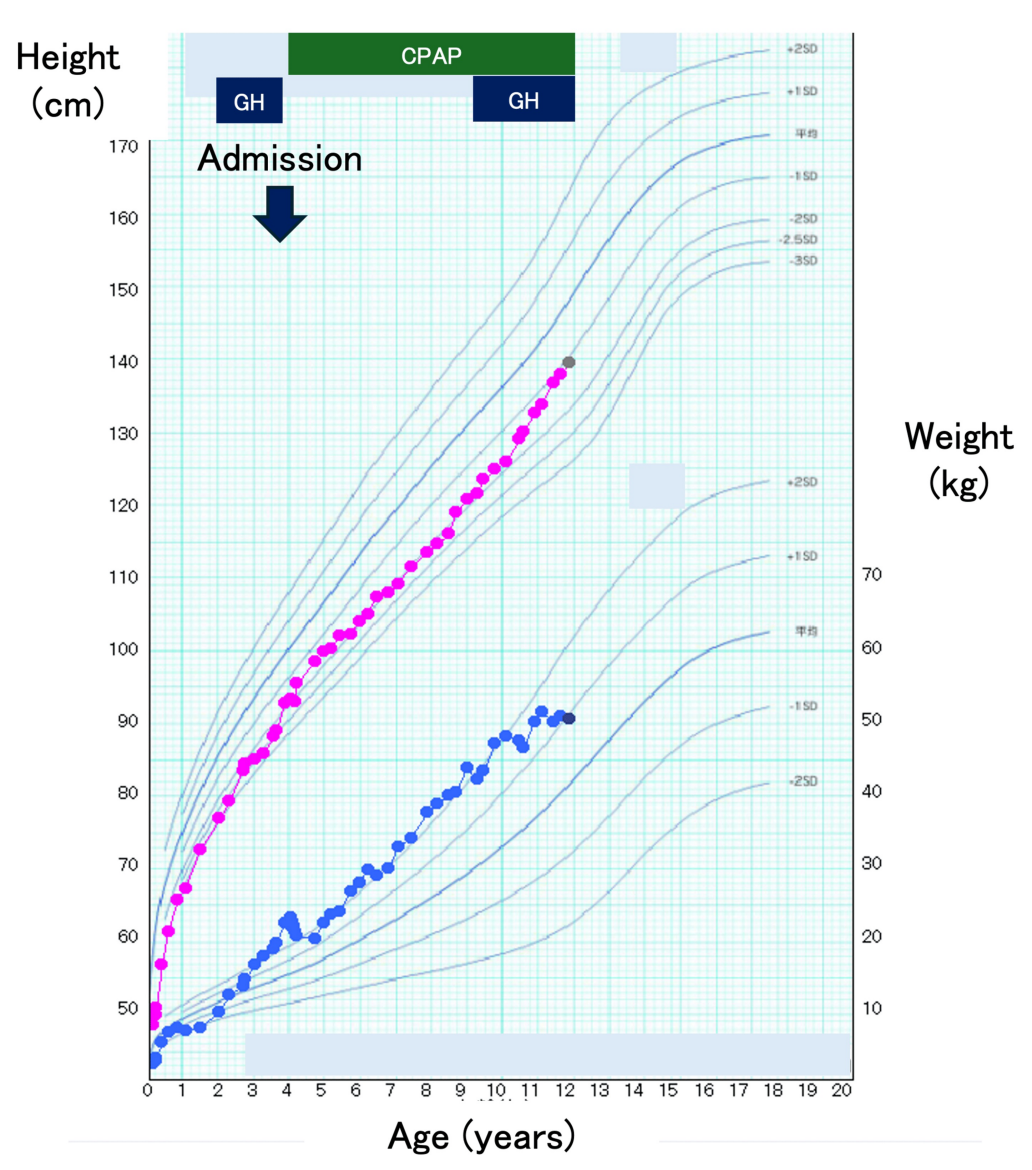
Growth trajectory and clinical course Height and weight are plotted on Japanese reference growth charts using The Cross-Sectional Growth Chart for Boys (The 2000 National Growth Survey on Preschool Children and School Health Statistics Research) [9].  Blue bars indicate growth hormone (GH) therapy, and the green bar indicates home continuous positive airway pressure therapy. The arrow marks hospitalization at four years of age owing to respiratory syncytial virus infection.

He underwent orchiopexy at two years and nine months of age. Recombinant human growth hormone (GH) therapy was initiated at three years and seven months of age (Figure 2) [9]. Formal respiratory evaluation, including polysomnography or echocardiography, had not been performed prior to this episode.

He developed rhinorrhea and cough on day 1 of illness. On day 2, during a routine visit to a rehabilitation facility, he had labored breathing and cyanosis and was transported to a secondary hospital. On arrival, he had fever (38.8°C), wheezing, and chest retractions, with oxygen saturation in the 80% range. Upper airway obstruction (including epiglottitis) was suspected, and dexamethasone and nebulized epinephrine were administered. Laboratory testing showed respiratory acidosis and markedly elevated liver enzymes. Therefore, he was transferred to our institution by helicopter for intensive management.

Findings on admission

The patient’s height was 93.0 cm (−2.10 standard deviation, SD), weight was 22.2 kg (+2.36 SD), obesity index was 64.4%, and body mass index was 25.7 (+4.64 SD). His temperature was 38.1°C, respiratory rate was 36 breaths/min, heart rate was 150 beats/min, and blood pressure was 111/58 mmHg. He required oxygen via a mask at 10 L/min and maintained SpO₂ at 98%-99%. Cardiac auscultation showed a holosystolic murmur. A lung exam showed crackles and increased work of breathing. The liver was palpable 3 cm below the costal margin. Laboratory findings on admission are summarized in Table 1, showing marked leukocytosis, severe hypertransaminasemia, coagulopathy, and evidence of acute systemic stress. Rapid testing was RSV-positive and influenza-negative. Arterial blood gas analysis revealed respiratory acidosis with hypercapnia and elevated lactate levels. 

**Table 1:** Laboratory findings on admission Laboratory evaluation on admission demonstrated severe hypertransaminasemia with profound elevation of aspartate aminotransferase (AST), alanine aminotransferase (ALT), and lactate dehydrogenase (LDH). Blood gas analysis revealed respiratory acidosis with hypercapnia. Alkaline phosphatase (ALP) reference ranges are based on normal pediatric bone metabolism.

Category	Parameter	Value	Reference range
Complete blood count	White blood cells (/µL)	27,660	5,000–15,000
	Red blood cells (×10⁶/µL)	4.29	3.9–5.3
	Hemoglobin (g/dL)	12.0	11.5–14.5
	Platelets (×10⁴/µL)	23.4	15–40
	Neutrophils (%)	86.0	30–60
	Lymphocytes (%)	10.0	30–60
	Monocytes (%)	4.0	2–10
Biochemistry	Total protein (g/dL)	5.0	6.0–8.0
	Albumin (g/dL)	3.0	3.8–5.0
	Total bilirubin (mg/dL)	0.7	0.2–1.2
	AST (U/L)	7,220	<40
	ALT (U/L)	3,270	<40
	LDH (U/L)	10,980	120–240
	Alkaline phosphatase (U/L)	1,223	350–1,000
	γ-GTP (U/L)	95	<30
	Blood urea nitrogen (mg/dL)	31.2	7–20
	Creatinine (mg/dL)	0.86	0.2–0.5
	C-reactive protein (mg/dL)	0.46	<0.3
	Sodium (mmol/L)	139	135–145
	Potassium (mmol/L)	4.3	3.5–5.0
	Chloride (mmol/L)	103	98–108
	IgG (mg/dL)	401	650–1,500
	IgM (mg/dL)	63	40–200
Blood gas analysis	pH	7.251	7.35–7.45
Arterial Blood	pCO₂ (mmHg)	53	35–45
(on oxygen at 10 L/min)	pO₂ (mmHg)	110	80–100
	HCO₃⁻ (mmol/L)	22.5	22–26
	Base excess (mmol/L)	−3.7	−2 to +2
	Lactate (mmol/L)	3.8	0.5–2.0
Coagulation	Prothrombin time (sec)	35.2	10–14
	Prothrombin time activity (%)	18.8	70–120
	PT-INR	2.78	0.9–1.1
	Activated partial thromboplastin time (sec)	36.3	25–35
	Fibrinogen (mg/dL)	173	200–400
Microbiology	Respiratory syncytial virus	Positive	Negative
	Influenza virus	Negative	Negative

**Figure 3:**
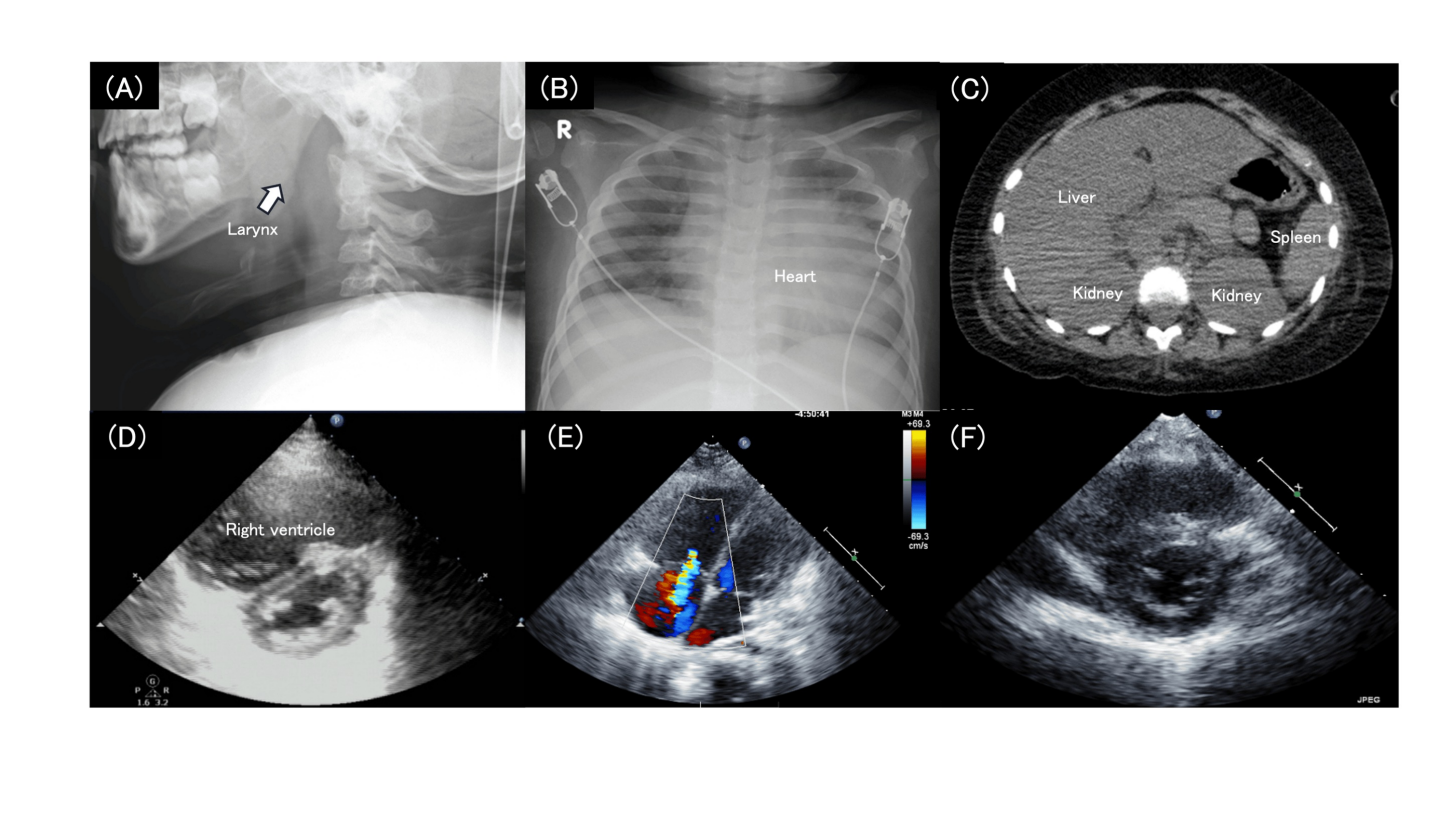
Imaging findings during acute respiratory decompensation (A) Lateral neck radiograph demonstrating narrowing of the upper airway at the laryngeal level, (B) Chest radiograph showing bilateral decreased lung transparency and cardiomegaly with a cardiothoracic ratio of 0.68, (C) Abdominal computed tomography demonstrating hepatomegaly with increased hepatic attenuation, (D, E) Echocardiography at admission demonstrated marked right ventricular hypertrophy resulting in a D-shaped left ventricle. Tricuspid regurgitation was also observed, suggesting the presence of pulmonary hypertension, (F) Follow-up echocardiography one month later showed improvement in right ventricular dilatation and resolution of left ventricular compression.

An X-ray showed laryngeal obstruction, lung consolidation, and cardiomegaly, with an increased cardiothoracic ratio of 0.68 (Figure 3 ). Echocardiography demonstrated marked right ventricular dilatation, consistent with pulmonary hypertension-related acute right ventricular pressure overload (Figure 3C). Quantitative echocardiographic assessment revealed an acceleration-time to ejection-time ratio (AcT/ET) of 118/300 ms (0.39), a tricuspid regurgitation (TR) velocity of 3.3 m/s with an estimated pressure gradient (PG) of 44 mmHg. The right ventricular pressure was estimated to be approximately 40% of the left ventricular pressure (RVP/LVP = 0.4), supporting the presence of moderate pulmonary hypertension. Abdominal computed tomography showed hepatomegaly with increased hepatic attenuation, which suggested congestive hepatopathy secondary to right-sided heart failure (Figure 3D).

Clinical course

The patient was admitted to the intensive care unit, where respiratory support and comprehensive systemic management were initiated. Noninvasive ventilation (bilevel positive airway pressure) was initiated on admission. Diuretic therapy (furosemide) was started because of right-sided cardiac dilation on echocardiography. He was gradually stabilized and transitioned from bilevel positive airway pressure to nasal continuous positive airway pressure (CPAP) on hospital day 6. Daytime supplemental oxygen was discontinued on day 12, and nocturnal CPAP alone was continued. As his respiratory status improved, aminotransferase levels returned to normal, which was consistent with reversible congestive hepatopathy and/or hypoxic/ischemic liver injury in the setting of cardiopulmonary decompensation.

**Figure 4:**
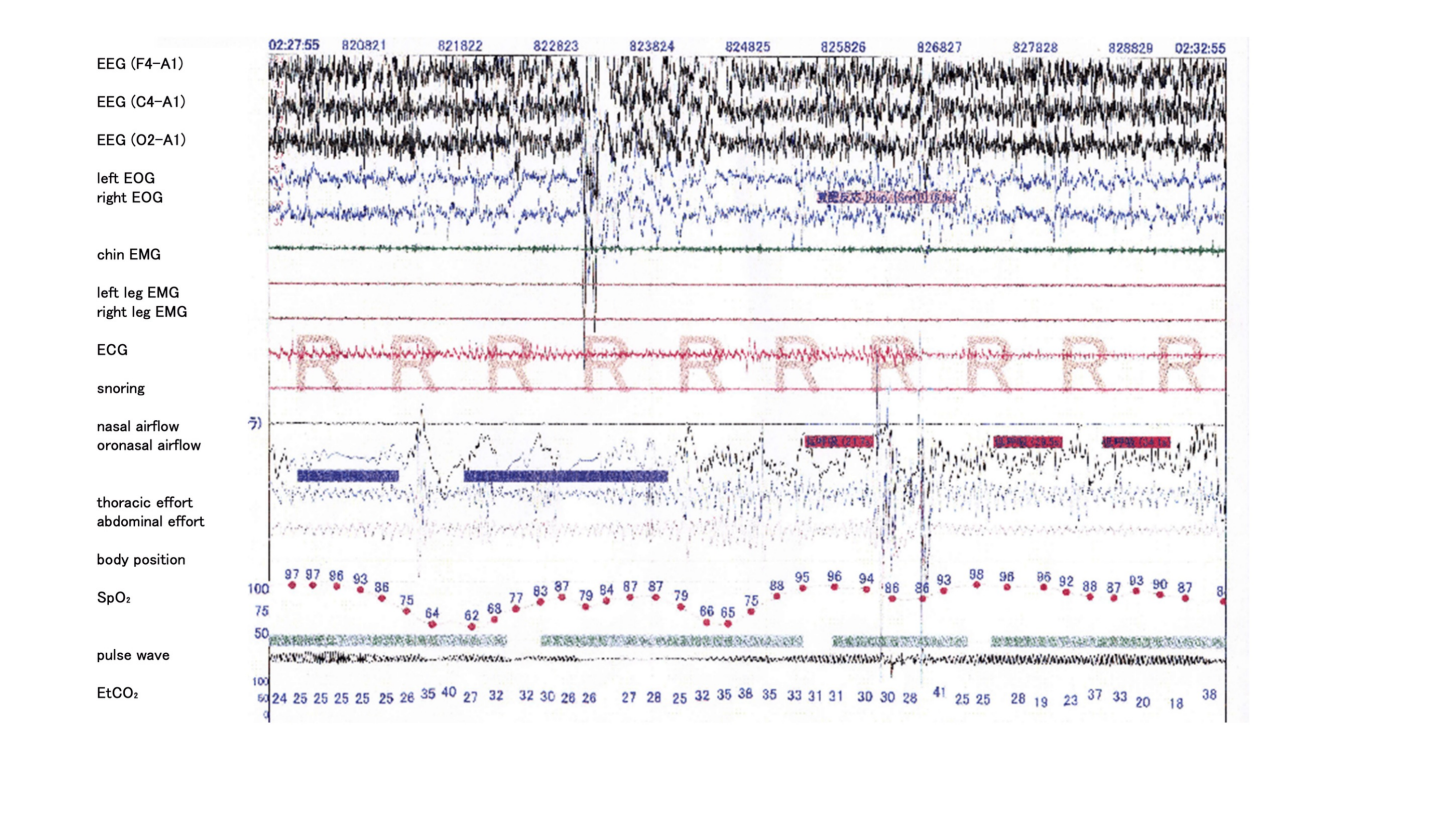
Overnight polysomnographic findings Overnight polysomnography (PSG) performed after respiratory stabilization showed sleep-disordered breathing. The apnea–hypopnea index (AHI) was 10.8 events/h, consisting predominantly of hypopneas (hypopnea index, 10.2 events/h), with a low apnea index of 0.6 events/h. Marked nocturnal oxygen desaturation was observed, with a minimum oxygen saturation (SpO₂ nadir) of 52%.

During hospitalization, a caloric-restricted diet (approximately 45 kcal/kg/day) was implemented with parents' education. GH therapy was temporarily discontinued. Serial measurements demonstrated gradual normalization of aminotransferase levels. Before discharge, overnight polysomnography showed clinically significant SDB, with an apnea-hypopnea index of 10.8 events/h (predominantly hypopneas), obstructive apneas, and profound nocturnal oxygen desaturation (SpO₂ nadir, 52%). This situation led to the initiation of home continuous positive airway pressure therapy (Figure 4). Follow-up echocardiography performed six months after discharge demonstrated improvement in right ventricular size; the AcT/ET was 92/327 ms (0.28), and the TR velocity was 2.1 m/s with an estimated PG of 17 mmHg, indicating resolution of pulmonary hypertension. GH therapy was later restarted at nine years of age after improvement in the severity of obesity and the sleep status (Figure 1).

## Discussion

This case illustrates how RSV infection can precipitate rapid cardiopulmonary decompensation in a child with PWS who likely had pre-existing vulnerability from obesity, hypotonia, and unrecognized sleep-related airway obstruction and nocturnal hypoxemia. Children with PWS frequently show abnormal ventilatory control and impaired arousal to hypoxemia or hypercapnia, together with upper airway narrowing and obesity-related mechanical load, predisposing them to OSA and hypoventilation [2,3]. In such vulnerable populations, RSV-induced hypoxemia can provoke hypoxic pulmonary vasoconstriction, leading to an acute increase in pulmonary vascular resistance and, in severe cases, a pulmonary hypertensive crisis with right ventricular dysfunction [7,8]. In our patient, RSV lower respiratory tract infection likely increased airway resistance, worsened ventilation-perfusion mismatch, and amplified nocturnal hypoxemia. Acute hypoxemia can induce hypoxic pulmonary vasoconstriction, thereby increasing pulmonary vascular resistance and right ventricular afterload. In susceptible patients, this series of events may result in pulmonary hypertension, right-sided dilation, and cor pulmonale. Respiratory failure and obesity-related cor pulmonale have been repeatedly implicated among severe and fatal events in PWS. 

The marked elevation in transaminases with coagulopathy in this patient promptly improved with cardiopulmonary stabilization, which suggested reversible liver injury related to acute hemodynamic compromise, congestive hepatopathy, and ischemic hepatitis [10]. While ischemic hepatitis classically occurs with systemic hypotension, severe hypoxemia, and right heart failure can also impair hepatic oxygen delivery and venous drainage, leading to an elevation in aminotransferases [11].

GH therapy is essential in managing PWS, including in our patient. However, several reports have emphasized careful respiratory evaluation, including polysomnographic assessment, at baseline and during follow-up for PWS [5,6]. SDB can evolve over time and may worsen after GH therapy in PWS. Our patient’s polysomnography showed a modestapnea-hypopnea index but profound desaturation (SpO₂ nadir, 52%). These findings suggest that clinical severity in PWS may not be captured by the apnea-hypopnea index alone; sustained hypoxemia and hypoventilation components should be assessed, and noninvasive ventilation strategies should be individualized.

Palivizumab prophylaxis reduces hospitalization for RSV in infants and young children at increased risk, including those with hemodynamically significant congenital heart disease [12]. Nirsevimab has also recently emerged as an effective RSV preventive strategy option for high-risk patients [13]. The American Academy of Pediatrics guidelines emphasize targeted prophylaxis for defined high-risk groups. These guidelines also note that clinicians may consider prophylaxis for children with pulmonary abnormalities or neuromuscular diseases that impair airway clearance, recognizing that such children can have severe outcomes from RSV infection [14]. Many young children with PWS have severe hypotonia and impaired airway clearance. On the basis of current evidence, individualized consideration may be reasonable for selected young patients with PWS who have hypotonia with considerable sleep-related hypoventilation with cardiopulmonary consequences. Future studies are required to define risk stratification and cost-effectiveness of RSV immunoprophylaxis in PWS.

## Conclusions

RSV infection can trigger acute respiratory failure with pulmonary hypertension, cor pulmonale, and secondary liver injury in children with PWS, particularly when severe nocturnal hypoxemia or OSA is unrecognized. Systematic screening and longitudinal surveillance for SDB and cardiopulmonary complications are essential in PWS, especially in the context of obesity and GH therapy. RSV prophylaxis with palivizumab or nirsevimab may merit individualized consideration for high-risk young children with PWS who have impaired airway clearance or considerable respiratory comorbidity.
